# Towards a partial order graph for interactive pharmacophore exploration: extraction of pharmacophores activity delta

**DOI:** 10.1186/s13321-023-00782-0

**Published:** 2023-11-29

**Authors:** Etienne Lehembre, Johanna Giovannini, Damien Geslin, Alban Lepailleur, Jean-Luc Lamotte, David Auber, Abdelkader Ouali, Bruno Cremilleux, Albrecht Zimmermann, Bertrand Cuissart, Ronan Bureau

**Affiliations:** 1 Centre d’Etudes Et de Recherche Sur Le Médicament de Normandie, Normandie Université, UNICAEN, CERMN, 14000 Caen, France; 2 Groupe de Recherche en Informatique, Image, Automatique Et Instrumentation de Caen, Normandie Université, UNICAEN, ENSICAEN, CNRS, GREYC, 14000 Caen, France; 3 Univ. Bordeaux, CNRS, Bordeaux INP, INRIA, LaBRI, Talence, France

**Keywords:** Hasse diagram, Partial order graph, Pharmacophore, BCR-ABL, Siblings, Activity delta

## Abstract

This paper presents a novel approach called Pharmacophore Activity Delta for extracting outstanding pharmacophores from a chemogenomic dataset, with a specific focus on a kinase target known as BCR-ABL. The method involves constructing a Hasse diagram, referred to as the pharmacophore network, by utilizing the subgraph partial order as an initial step, leading to the identification of pharmacophores for further evaluation. A pharmacophore is classified as a ‘Pharmacophore Activity Delta’ if its capability to effectively discriminate between active vs inactive molecules significantly deviates (by at least δ standard deviations) from the mean capability of its related pharmacophores. Among the 1479 molecules associated to BCR-ABL binding data, 130 Pharmacophore Activity Delta were identified. The pharmacophore network reveals distinct regions associated with active and inactive molecules. The study includes a discussion on representative key areas linked to different pharmacophores, emphasizing structure–activity relationships.

## Introduction

The investigation of structure–activity relationships (Structure–Activity Relationships, SAR: relationship between the structures of chemicals and their biological activities) represents one of the most important tasks during the early stages of the drug discovery process [1]. The definition of pharmacophores as a key to drug design is very well accepted in the field of medicinal chemistry and is a key point to understand a molecule’s affinity for a biological receptor [2]. In our initial publication on topological pharmacophores [3], we described the logic for the definition of a new type of descriptor based on the notion of emergent pharmacophores. We repeat some points here to clarify the objectives of this work.

A pharmacophore corresponds to the greatest common structural denominator associated with a group of compounds exhibiting the same biological response profile [4]. Given a specific target, ligand-based pharmacophore elucidation requires the detection of the spatial arrangement of a combination of chemical features shared by several active molecules and responsible for favorable interactions with the active site. To discover these common anchoring features, the usual method starts with a careful selection of a small subset of ligands known for binding to the same active site with the same binding mode [5]. We have done several studies with this approach (see [6] for an example).

In recent years, the integration of large chemical databases [7] into the definition of SARs has been clearly explored. With SARs and pharmacophores in mind, we have introduced a method that automatically computes pharmacophores from a large data set of molecules without any prior supervised selection of a small subset of molecules [3]. That method was based on the computation of the so-called topological pharmacophores [8, 9].

Considering graph theory, 2D topological pharmacophores represent patterns which are present in a number of chemical structures. When applied to a data set partitioned into two classes (e.g., active vs inactive molecules), emerging pattern mining can identify the patterns that occur with higher frequency in one of the two classes [3].

Of these topological pharmacophores, we can highlight those associated with particular properties. We have previously explored a selection based on a growth rate value called GR (Growth rate, GR: ratio of frequencies of appearance of a pharmacophore in a given class of molecules compared to the other class (active or inactive compounds on BCR-ABL)). It corresponds to the frequency of appearance of a pharmacophore in one class (active, for example) compared to another. An initial selection was based on a value of 3 for the GR (ratio of 3:1 for the frequencies between the two groups). A technique named Maximal Marginal Relevance Feature Selection (Maximal Marginal Relevance Feature Selection, MMRFS: selection of relevant pharmacophores by considering their number of associated chemicals and their GR values) [10] has also allowed us to select a restricted subset of these topological pharmacophores. This subset keeps the same statistical performance as the complete set (sensitivity/specificity) with equivalent coverage of the compounds. First, pharmacophore networks were defined based on these subsets by considering a graph editing distance [11] for the calculation of the similarity between MMRFS pharmacophores and clustering techniques [12]. For SAR studies and only for this objective, we have thought of inverting the frequencies (inactive vs active) and thus characterized topological pharmacophores associated with inactive compounds. This gave us new insights into our data even if we are far from the historical definition of pharmacophores.

In this study, we have chosen to focus on another view of our topological pharmacophores with the definition of outstanding pharmacophores named Pharmacophore Activity Delta (Pharmacophore Activity Delta, PAD: Pharmacophore for which the discrimination between active vs inactive molecules significantly deviates from the mean capability of its related pharmacophores.). To find these PADs, a Hasse diagram [13, 14] was defined as a representation of the set of pharmacophores. This Hasse diagram corresponds to a partial order graph [14, 15] encoding a partial order between pharmacophores, also called a pharmacophore network. In this work, we leverage the pharmacophore network to quickly obtain the siblings of a given pharmacophore.

For each pharmacophore, we quantify its level of significance using a quality measure function, assigning a real number to each pharmacophore. We focus on the ratio of active molecules with respect to a specific receptor, making the growth rate one of the functions used to assess the quality of our pharmacophores. Pharmacophores that score very differently than the average of neighboring pharmacophores are considered to be PADs. The definition of the neighbors is based on the notion of siblings related to the Hasse diagram (vide infra). Using the growth rate, we show experimentally that very few patterns turn out to be PADs.

## Methods

### Dataset

In line with our previous paper [12], we retrieved a ChEMBL compound data set of BCR-ABL ligands [16–18] (target ChEMBL ID: CHEMBL1862, ChEMBL24 [19]). After discarding compounds with molecular weight above or equal to 800 g/mol, we obtained a data set of 1479 molecules with either K_i_ or IC_50_ information. This limitation is primarily associated with the combinatorial challenge when dealing with a molecule with a significant number of pharmacophoric functions (vide infra). Of these 1479 molecules, 773 were designated active compounds (meaning their K_i_ or IC_50_ value was below or equal to 100 nM).

### Pharmacophores

In agreement with our previous description for the generation of pharmacophores [3, 12], the pharmacophoric features correspond to generalized functionalities that are involved in favorable interactions between ligands and targets, including hydrogen-bond acceptors (|A|) and donors (|D|), negatively (|N|) and positively (|P|) charged ionizable groups, hydrophobic regions (|H|), and aromatic rings (|R|). Therefore, a pharmacophore is a fully connected graph where each vertex represents one of the specific pharmacophoric features, and the edges are labeled with the number of the fewest possible bonds between two vertices. The number of vertices, i.e. pharmacophoric features, composing a pharmacophore is called its order.

A notation was fixed for the pharmacophores. We started with the vertex of the pharmacophores, e.g., |A|A| for a pharmacophore with two As with pipes as separators, and we indicated the values of the edges, e.g., |2| for a distance of 2 bonds between the As, with pipes as separators (final notation: |A|A| |2|). For a more complex case with four pharmacophoric features and six distances to integrate, for instance |A|A|H|D| |2|4|5|7|1|3|, the six distances correspond to the first one against the others (|A|A|, |A|H|, |A|D|) then, the second one against the others (|A|H|, |A|D|) and, at the end, the last one against the other (|H|D|). In the following, we omit edges information and “|” separators in figures when they are not necessary for comprehension.

We call “support” the set of molecules supporting a given topological pharmacophore, i.e., containing all the pharmacophoric features of the pharmacophore with the correct distances between them. Let $p$ a pharmacophore and $D$ the set of studied molecules. We note $Support(p)$ the support of $p$ in $D$, i.e., its set of supporting molecules.

In agreement with our previous studies [3, 12], the minimal support for the extraction of pharmacophores was fixed to 10 (minimal number of compounds), and the orders (number of pharmacophoric features) were between 1 and 7. 112 291 pharmacophores were generated with these parameters. For the GR calculation (vide infra), the cutoff for active derivatives was fixed to be less than or equal to 100 nM (773 compounds).

Let $p,q$ be two 2D pharmacophores assimilated to graphs with labeled vertices and labeled edges and *D* the set of studied molecules. If $p$ is a subgraph of $q$, noted $p \subset q$, this means that the pharmacophore $p$ is included in the pharmacophore $q$. It also means that every single molecule covered by $q$ is also covered by $p$. A molecule set covered by a pharmacophore $p$ is the support of a pharmacophore denoted $\mathrm{Support} (p)\subset D$. Thus, we can state that $p \subset q$ implies $Support (q)\subset Support (p)$.

From the subgraph partial order we can build a Hasse diagram [20] called a pharmacophore network. We note $G (V,E)$ a pharmacophore network where each vertex $v \in V$ is a pharmacophore and given two vertices ${v}_{1},{v}_{2}\in V,$ $\exists \left({v}_{1},{v}_{2}\right)\in E$ ($E$ is the set of edges between the pharmacophore network vertices) if and only if ${v}_{1} \subset {v}_{2}$ and $\nexists {v}_{3} \in V$ such that ${v}_{1}\subset {v}_{3}$ and ${v}_{3} \subset {v}_{2}$. Therefore, an edge links two vertices of the network ${v}_{1},{v}_{2}\in V$ if and only if the pharmacophore in ${v}_{1}$ is a subgraph of the pharmacophore contained in ${v}_{2}$ and there are no pharmacophores ${v}_{3}$ in the pharmacophore network subgraph of ${v}_{2}$ which has ${v}_{1}$ as subgraph.

**Fig. 1:**
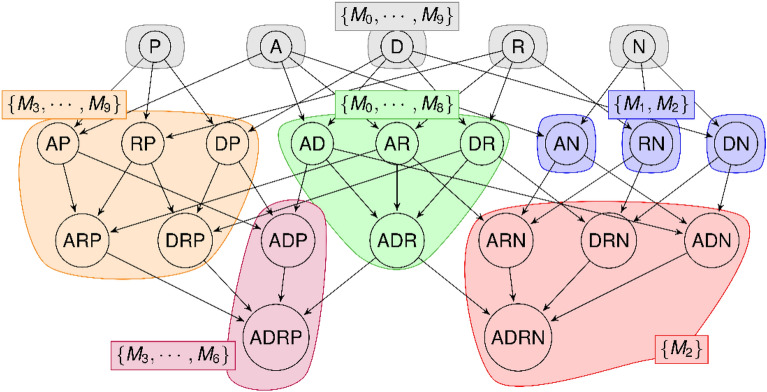
Structure of the pharmacophore network. Each circle is a vertex containing a pharmacophore. Only the pharmacophoric features are displayed to simplify the example and the separators “|” are removed for ease of readability. Molecules having the pharmacophore are indicated in the colored rectangles using set notation. The notation $\{{M}_{3},\dots ,{M}_{6}\}$ indicates that the set is composed of molecules ${M}_{3},{M}_{4},{M}_{5},and\,{M}_{6}$. The molecules associated to a pharmacophore is determined by the colored area its vertex is in. Edges displays the inclusion relation between pharmacophores. The vertex containing AN is connected to the vertices containing ARN and ADN because AN is a subgraph of ARN and ADN. Since AN is associated with molecules ${M}_{1}$ and ${M}_{2}$, ARN and ADN must be associated to a subset of ${\{M}_{1},{M}_{2}\}$. In this example, these pharmacophores are associated to the molecule ${M}_{2}$.

We note the edge relation between the vertices of the pharmacophore network ${v}_{1}< {v}_{2}$ and call ${v}_{1}$ a parent, which means that ${v}_{2}$ is called a child. It also means that $Support\left({v}_{2}\right)\subset Support\left({v}_{1}\right)$. We illustrate the obtained structure in Fig. 1.

As we noticed that a large number of pharmacophores appear in the exact same set of molecules, we decided to group them into equivalence classes [21] (ECs) based on molecule sets.

### GEC, DEC, SEC

The first one is the General Equivalence Class (GEC), which groups every pharmacophore covering the same set of molecules. Let $p$ a pharmacophore and $G(V, E)$ a pharmacophore network containing $p$, its general equivalence class is defined as $GEC\left(p,G\right)=\left\{v\in V | Support\left(v\right)=Support\left(p\right)\right\}$. The formula can be transcribed as follows. Given a pharmacophore $p$ and a graph $G$, the general equivalence class of $p$ is the set of pharmacophores $v$ contained in the vertices $V$ of $G$ having the same support as $p$, i.e. associated to the same set of molecules. In Fig. 1**,** these equivalence classes are indicated by the colors of the areas. Meaning that pharmacophores of the first layer belong to the same general equivalence class because they all are in grey areas.

The second one is the Divided Equivalence Class (DEC), which groups every pharmacophore that has the same set of molecules and the same order. Let p a pharmacophore and $G(V, E)$ a pharmacophore network containing $p$, we label Order(p) its number of pharmacophoric features. Then, the divided equivalence class of $p$ is defined as $DEC\left(p,G\right)=\left\{v\in GEC\left(p,G\right) | Order\left(v\right)=Order\left(p\right)\right\}$. The formula can be transcribed as follows. Given a pharmacophore $p$ and a graph $G$, the divided equivalence class of $p$ is the set of pharmacophores contained in the general equivalence class of $p$ in $G$ having the same order, i.e., having the same number of pharmacophoric features. In Fig. 1, pharmacophores in the orange area belong to the same general equivalence class but are divided in two divided equivalence class regarding the layer they belong to, i.e., regarding their orders.

The last one is a specialization of GECs based on the connectivity of the pharmacophores in the pharmacophore network called the Structured Equivalence Class (SEC). To define this class, we introduce a new operator. Let $p,v$ two pharmacophores in the vertices of the pharmacophore network $G\left(V,E\right)$; we note $p \sim v$ if we have $p < v$ or $v < p$. Thus, given ${v}_{1},\dots ,{v}_{n}\in V$, the expression $\left(p \sim {v}_{1} \sim \cdots \sim {v}_{n} \sim v\right)$ indicates that a path exists in the pharmacophore network going from the vertex $p$ to the vertex $v$. A structured equivalence class groups all pharmacophores occurring in the same set of molecules having a path connecting them inside their GEC. Let $p$ a pharmacophore, its structured equivalence class is defined as $SEC\left(p,G\right)=\{v\in GEC\left(p,G\right) | \left(\left(p \sim v\right), or \left(\exists {v}_{1},\cdots , {v}_{n} \in GEC\left(p,G\right), \left(p \sim {v}_{1} \sim \cdots \sim {v}_{n} \sim v\right)\right)\right)\}$. The formula can be transcribed as follows. Given a pharmacophore $p$ and a graph $G$, the structured equivalence class of $p$ is the set of pharmacophores $v$ in $V$ contained in the general equivalence class of $p$ in $G$ which are connected to $p$ by a path only visiting pharmacophores contained in the general equivalence class of $p$ in $G$. In Fig. 1**,** the pharmacophores in the grey areas all belong to the same general equivalence class but they all belong to separated structure equivalence classes.

The concepts of GEC, DEC and SEC all fall under a common concept called Equivalence Classes (EC). We can construct a pharmacophore network that minimizes redundant information from the ECs within a given pharmacophore network by taking ECs as vertices and extending the partial order as follows. Let $E{C}_{1},E{C}_{2}$ be two equivalence classes; we say that $E{C}_{1}<E{C}_{2}$ if and only if $\exists {e}_{1}\in E{C}_{1},\exists {e}_{2}\in E{C}_{2},{e}_{1}<{e}_{2}$. With the extended partial order, we can define a pharmacophore network of equivalence classes following the same principles as the one used to compute the ECs. Below, we introduce methods applied to the pharmacophore network. These methods can be applied to either pharmacophores as vertices or equivalence classes as vertices. We refer to it as the GEC (respectively DEC and SEC) network when the vertices of the network are general (respectively divided and structured) equivalence classes.

In Fig. 1, the three pharmacophores appearing only in the molecule ${M}_{1}$ and ${M}_{2}$ (in the blue areas) belongs to the same GEC and DEC, but do not belong to the same SEC because they are not connected within their GEC, *i.e*., the path linking one to another in the context graph has to go through a vertex which covers different molecules set. But if you consider pharmacophores, ADN, DNR and ARN, they belong to the same SEC (the purple area) because ADRN is associated with the same set of molecules. As we can observe, the set inclusion of molecules is maintained, which indicates that there is an equivalence between the two types of pharmacophore network.

**Fig. 2:**
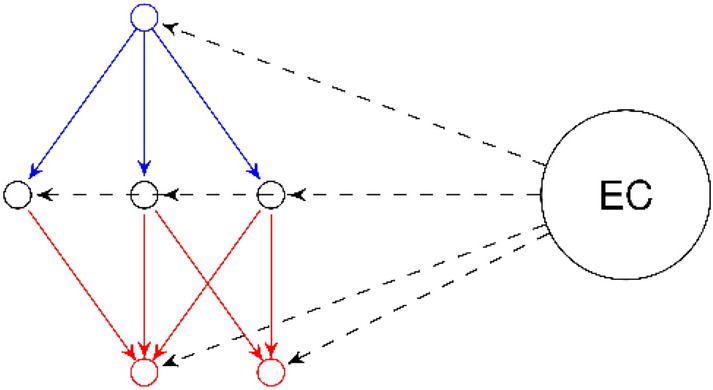
Generating pharmacophore (blue) and closed pharmacophores (red) from one EC (right)

In order to study the equivalence classes, without considering every redundant pharmacophore contained, we use the notion of generating pharmacophores called generators and closed pharmacophores. Generators are pharmacophores that have no parents in their Equivalence Class (EC), which means they are the starting points of the EC. Closed pharmacophores are pharmacophores that have no children in their EC, which means they are the endpoints of their EC. In Fig. 2, each circle without label is a pharmacophore (left) contained in the circle labeled $EC$ which is an equivalence class (right). Dashed lines symbolize the inclusion relation between the pharmacophores and the equivalence class. We have one generator in blue and two closed pharmacophores in red.

But even with the use of equivalence classes, there are still too many vertices to study in the pharmacophore network. Therefore, we use the notion of siblings in a pharmacophore network. From intuition, a sibling is a vertex having at least one common parent. For a given pharmacophore $p$ and its pharmacophore network $G\left(V,E\right)$ where each vertex $v \in V$ is a pharmacophore, the siblings set of $p$ is defined as ${S(p,G)=\{{v}_{1} \in V | \exists v}_{2} \in V, { v}_{2}< p \mathrm{ ~and~ }{ v}_{2}< {v}_{1} \}$. We note $Card(p, G)$ the cardinal of the set of siblings of $p$, i.e., the number of pharmacophores contained in the siblings set.

**Fig. 3:**
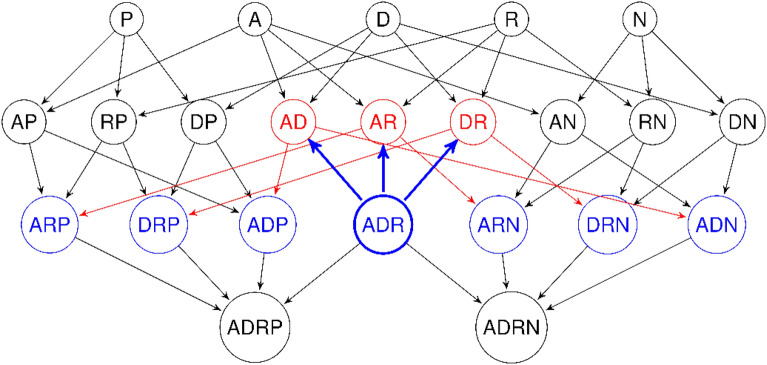
Getting the siblings ARP, DRP, ADP, ARN, DRN, and ADN (blue) from an origin vertex labeled *ADR* (bold blue); its parents are $AD, AR~and~ DR$ (red)

In the pharmacophore network (see Fig. 3), the siblings have at most one pharmacophoric feature which differs from the origin pharmacophore. In the condensed graph, the siblings cover the closest sets of molecules which are not included in one another because they all have molecule subsets of their common parents.

Using the concept of *siblings*, we will identify the $ECs$ whose quality strongly deviates from those of their siblings. We interpret those $EC$ s as key graph elements, as they may explain the biological behavior of their supporting molecules. We call in the following the selected outstanding pharmacophores the *Pharmacophore Activity Delta* (PAD).

### Pharmacophore activity delta

Let *p* a pharmacophore and $D$ the molecule data set. We call the quality of *p* a real number determined by a function considering the molecules containing *p* noted *f(p, D)*. In this work, the quality is the normalized growth rate of $p$. We say that a pharmacophore *p* is a PAD when its quality deviates from the mean quality of its siblings *S*(*p, G*). Let *f(p, D)* the quality measure’s value of the pharmacophore *p* in the dataset *D*, the sibling mean *µ*(*S*(*p, G*)*, D*) is:

1 $$\upmu \left(S\left(p, G\right),D\right)=\frac{{\sum }_{s\in S\left(p, G\right)}f\left(s,D\right)}{Card\left(S\left(p, G\right)\right)}$$

Then, *σ(S(*$p, G$*), D)* is defined as the standard deviation of the siblings:

2 $$\upsigma \left(S\left(p, G\right),D\right)=\sqrt{\frac{{\sum }_{s\in S\left(p\right)}{\left(f\left(s,D\right)-\upmu \left(S\left(p, G\right),D\right)\right)}^{2}}{Card\left(S\left(p, G\right)\right)}}$$

The pertinence of $p$ is defined as:

$$Pert\left(p,G,D\right)=\frac{f\left(p,D\right)-\upmu \left(S\left(p, G\right),D\right)}{\upsigma \left(S\left(p, G\right)\right)}$$

The pertinence is the deviation from the mean quality of the sibling divided by the standard deviation of the sibling. It can be transcribed as the deviation proportion of $p$ regarding it sibling. From this equation, a PAD is a pharmacophore which pertinence is high enough to interest the expert. Therefore, we define our PAD selector.

The selector is defined as:

3 $$PAD\left(\mathrm{G},f,D,\updelta \right)=\{p\in \mathrm{V} | \left|Pert\left(p,G,D\right)\right| \ge\updelta \}$$

Thus, a pharmacophore *p* is a PAD if its quality deviates at least *δ* standard deviations (Eq. 3) from the mean of the qualities of its siblings, *δ* being a user-supplied parameter.

We chose to use the standard deviation because we want to adapt our selection to each sibling. If the siblings are different from one another, we only want to select the one that deviates the most. If the siblings are similar to each other, then even a small deviation can be interesting.

### GR

Based on the partitioning of the initial dataset into active and inactive molecules (or the inverse) the growth rate (GR) of a given pharmacophore corresponds to the ratio between the frequencies with which it occurs in each of the two subgroups.

$$\mathrm{GR}=\frac{Fit \,frequency \,within \,actives}{Fit \,frequency \,within \,inactives}$$

The main metric in this study is GR_N_, normalized GR with values between 0 and 1.

$$G{R}_{N}=\frac{GR}{\left(GR+1\right)}$$

A GR value of 1 (same frequency for active and inactive compounds) corresponds to 0.5 for GR_N_. For the two extreme values, a GR_N_ value of 1 indicates that a pharmacophore occurs in only active compounds, and a value of 0, in only inactive compounds. A GR value of 3, classically used in our previous studies, now corresponds to a GR_N_ value of 0.75.

### Pharmacophore (and PAD) stability

Discovering interesting substructures from data always risks capturing spurious phenomena particular to the data set, instead of fundamental relationships that hold more generally. In the case of pharmacophore activity deltas, this risk is compounded by the fact that each PADs identification depends not only on its own support and quality, but also on those of its siblings (and, furthermore, on whether those siblings are present in the pharmacophore network at all).

To assess the stability of discovered PADs, we therefore use a ten-fold cross-validation of the data: the data set is split into ten equally-sized subsets (folds), which are then combined to derive ten subsets, each of which containing 90% of the whole data, keeping one fold apart each time. This allows to modify data sets in a controlled manner. PADs are identified independently on each of those 10 data sets, and we assess how often PAD (re)occurs in the different result sets.

Given the construction of the underlying data sets, any two such sets will share a proportion of about 1–10/90 = 0.88889 of the compounds. Simply based on this data overlap, we would expected particular pharmacophores to reoccur *k* times at most 0.88889^ k^ due to chance (e.g. 0.5549 for k = 5). As mentioned above, however, this probability will be significantly lower for PADs since not only their siblings need to reoccur but GR differences will also need to be large enough for a pharmacophore to be identified as a PAD.

## Results and discussion

### Pharmacophores and equivalence class network: GEC, DEC, SEC

**Fig. 4:**
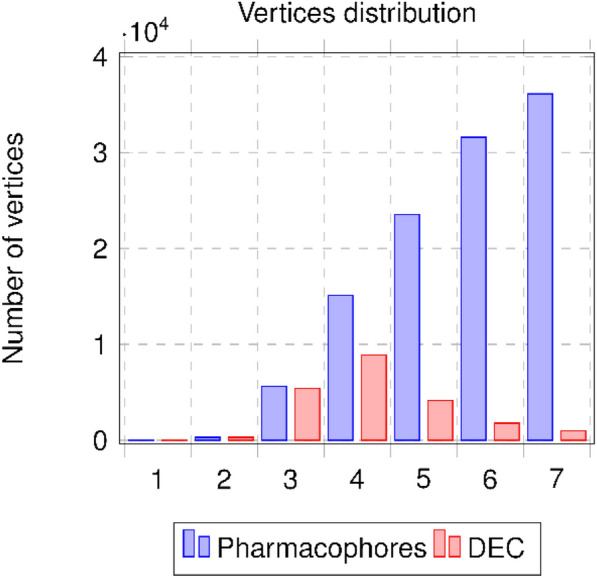
Distributions of vertices by order: initial pharmacophore network and DEC network

Figure 4 shows the initial pharmacophore network (blue) and the DEC network (red) illustrating the distribution of vertices regarding their layers. We can see that depending on the pharmacophore order, the number of DEC vertices is strongly reduced when the order increases. This phenomenon is predominant for orders 5, 6 and 7: those orders place a high number of pharmacophores into an equivalence class when considering the DEC definition. A multiplication of pharmacophores associated with the same set of molecules is clearly observed and amplified when the number of pharmacophoric features is integrated.

**Fig. 5:**
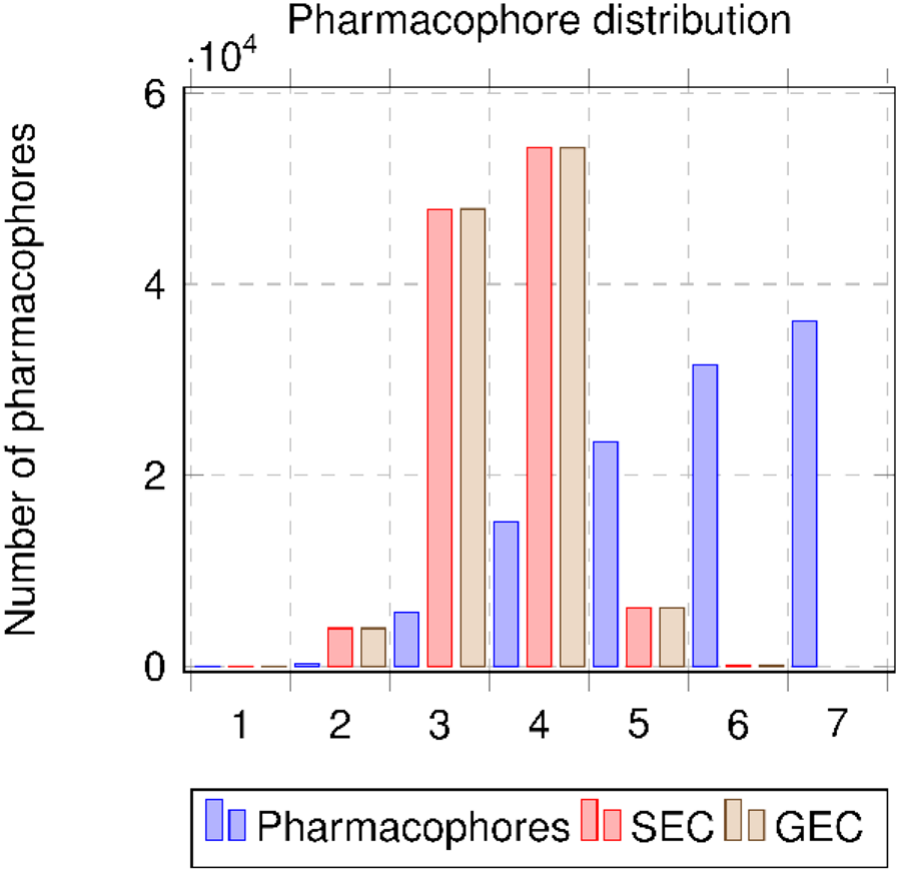
Distributions of pharmacophores by order for initial pharmacophore network (blue) and for GECs network (light brown) and SECs network (red) when considering the generators for the distributions

Figure 5 shows the pharmacophore distributions for the initial pharmacophore network (blue), the GEC network (light brown) and the SEC network (red). For each EC, we have kept the number of initial pharmacophores for a particular view of the modifications. The new distribution of pharmacophores through the notion of ECs in the order is based on the generators (smallest pharmacophore for each EC) for each EC. We can see clearly that the GECs and SECs have the same distributions and the pharmacophores of orders 5–7 are redistributed, through the generators, to orders 3 and 4.

**Fig. 6:**
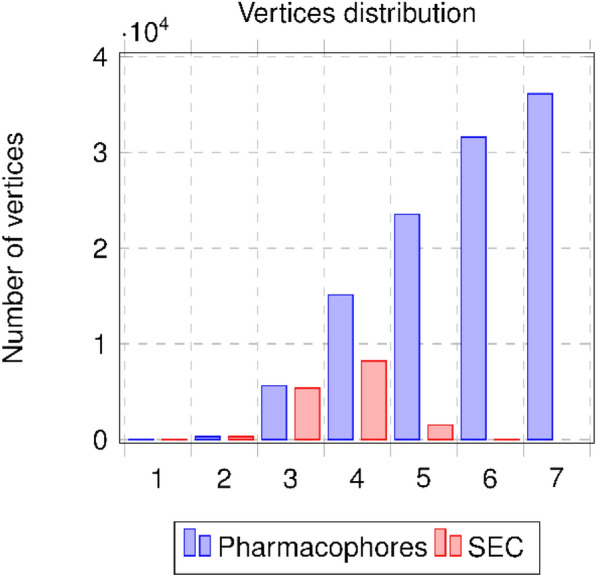
Distributions of vertices by order of pharmacophores for initial pharmacophore network (blue) and SECs network (red) by considering the generators for the distributions and one pharmacophore for each SEC

The last representation (see Fig. 6) shows the distribution of vertices in the initial pharmacophore network and in the SECs network by considering the order of the generators for each SEC. From 112 291 pharmacophores in our initial data set, we move to 15,477 SECs to be assessed.

### SEC/generators/parents

**Fig. 7:**
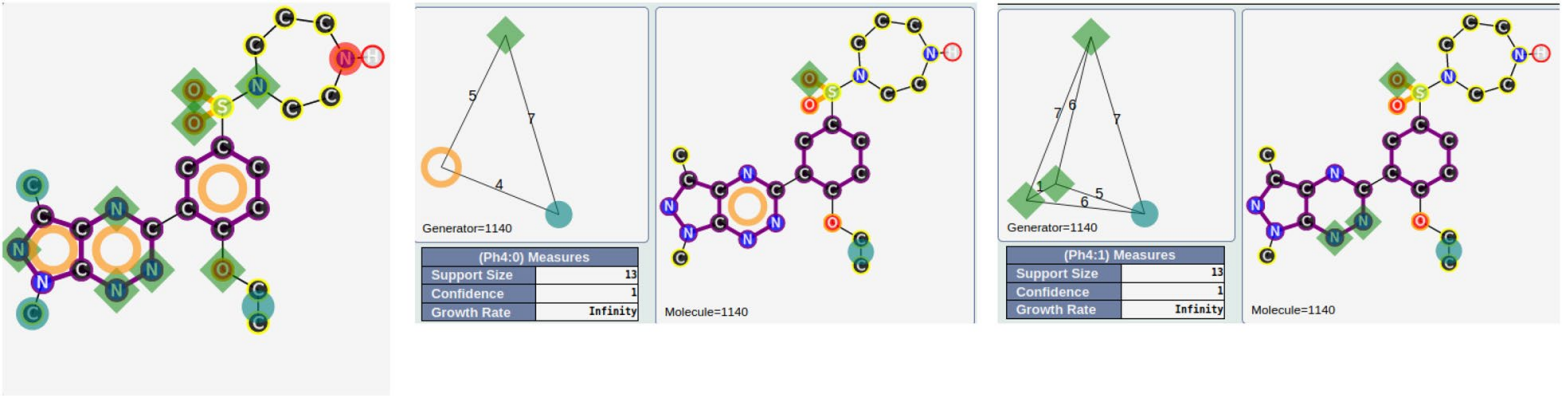
All pharmacophoric functions associated with one representative compound (left). Two among the 271 generators of this SEC (center and right)

Of the 15,477 SECs, 1745 are associated with at least two generators (vide supra for the definition). These 1745 SECs cover 1301 out of 1479 compounds. Of these 1745 SECs, 443 are associated with at least 5 generators, 25 with at least 30 generators, 10 with at least 50 generators and 1 with 271 generators. To give a first explanation of these results, the size of the molecules and the associated number of pharmacophoric functions were analyzed. In the initial dataset, 99 compounds have a molecular weight ≥ 500 g/mol and a number of pharmacophoric functions ≥ 20. Of these 99 compounds, 51 are associated with a SEC with at least 30 generators. So, the molecular weight and the number of associated pharmacophoric functions give a first and clear explanation for the observed number of generators associated with some SECs. The SEC with 271 generators corresponds to 13 compounds, all inactive (see Fig. 7 for illustrations of this SEC), compounds in agreement with the previous remark.

**Fig. 8:**
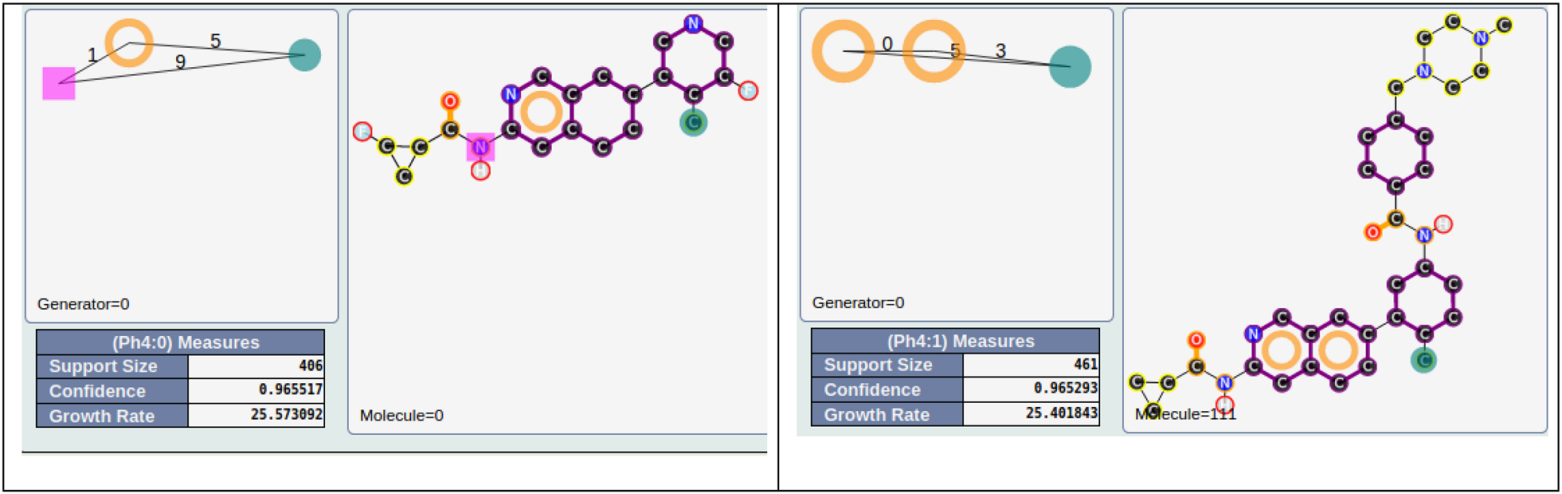
Best parents with more than 50 filiations

Starting from the 1745 previous SECs, we analyzed the parents of these SECs. We wanted to see if some parents have particular characteristics in terms of filiations. These 1745 SECs are associated with 7517 parents. Among them, 669 have more than 20 filiations and 201 have more than 50 filiations. Among the last group, 18 parents are associated with active compounds (GR_N_ ≥ 0.75) and 5 parents have a GR_N_ value ≥ 0.9. The best ones (w.r.t. GR_N_ values), |R|D|H| |1|5|9| and |R|R|H| |0|3|5|, are associated with 406 and 461 compounds, respectively (see Fig. 8). These pharmacophores correspond to important pharmacophores of this kinase with structural characteristics associated with the interaction with the hinge region and the key methyl group, often related to the back pocket region of the binding site (see Xing et al. [22] and our latest publication [12]).

Among the parents, the best one for the number of filiations, |A|R| |2|, has 276 filiations. It covers 1366 compounds out of 1475, with a GR_N_ value of 0.53.

### Outstanding pharmacophores: from SECs to PADs

With EP mining in mind, we applied the SEC network to our pharmacophore file and retrieved the GR_N_ values for each SEC. Table 1 shows information about the distribution of SECs as a function of the GR_N_ values. For the selection of PADs among the SECs, the pertinence value (Eq. 3, *δ* value) was considered first to be 1.96 (p-value of 0.05). 42 PADs (Table 1) were obtained, but with low coverage of the initial data set (22%). As a result, we have lowered the pertinence value to 1.64 (p-value of 0.1), and 377 PADs were obtained with a coverage of 81% of the initial data set (of the 277 compounds missing, 75 are actives).

**Table 1:** Description of SECs (as a function of GR_N_ values) and PADs (as a function of pertinence values)

Descriptions of SECs	Number
SECs: GR_N_ ≥ 0.5 / GR_N_ < 0.5	7534/7926 (SECs)
SECs: GR_N_ ≥ 0.75 / GR_N_ ≤ 0.25	4285/3803 (SECs)
SECs: GR_N_ = 1 / GR_N_ = 0	1084/979 (SECs)
Description of PADs	Number
PADs: pertinence ≥ 1.96 or ≤ -1.96 (α = 0.05)	20/22 (PADs)
Molecules covered (active/inactive)	337 (molecules) (187/150)
PADs: pertinence ≥ 1,64 or ≤ -1.64 (α = 0.1)	187/190 (PADs)
Molecules covered (active/inactive)	1202 (molecules) (698/504)
PADs MMRFS with α = 0.1	63 (0.85)/72 (0.60) (PADs (Recall values))
Molecules covered (active/inactive for above PADs MMRFS (// as separator))	659/44 // 19/426 (molecules)
Order 2 PADs	5/2 (PADs)
Order 3 PADs	45/56 (PADs)
Order 4 PADs	11/12 (PADs)
Order 5 PADs	2/2 (PADs)

Information on the associated number of SECs or PADs and the molecules covered for the PADs. Pertinence is related the Eq. 3

**Fig. 9:**
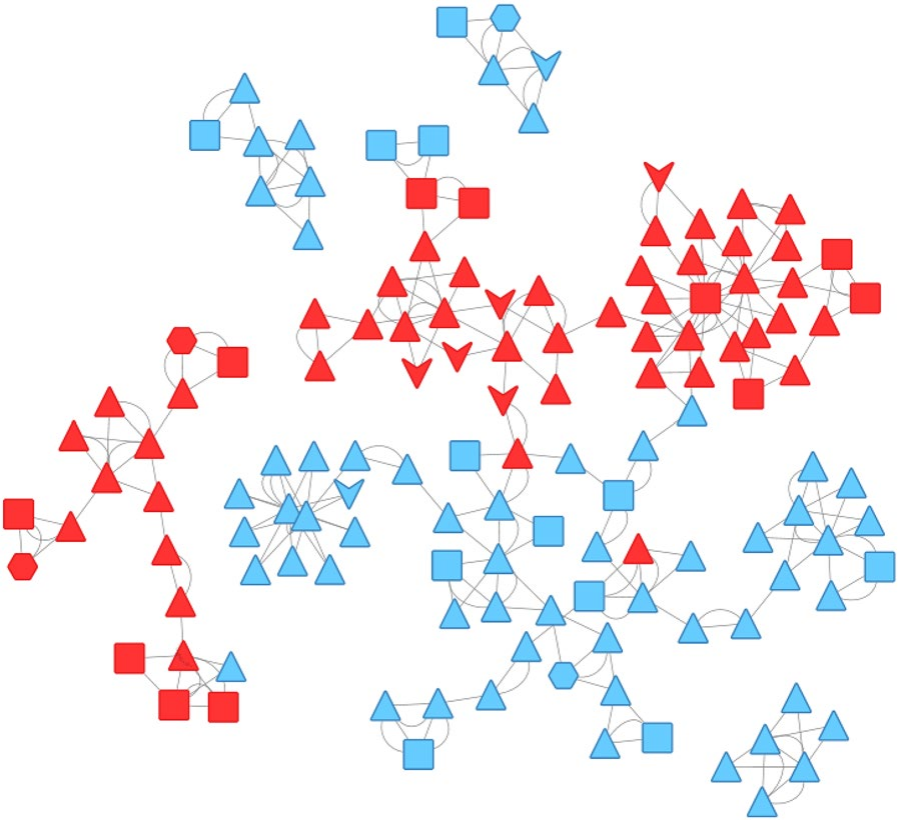
Pharmacophore Activity Delta network with active pharmacophores in red and inactive pharmacophores in cyan. The symbols are related to the order of the pharmacophores (order 2 (arrow), order 3 (triangle), order 4 (square), order 5 (hexagon)))

To analyze the PADs, we have chosen to represent them as a pharmacophore network. A similarity matrix was defined for the initial chemical data set with ECFP4 as molecular fingerprint descriptors. The Tanimoto coefficient was used as the similarity measure. The similarity between the PADs was defined as the average similarity between the molecules associated with the PADs. The orders of the PADs are different, so it was impossible to integrate a graph edit distance in agreement with our previous studies for the similarities between the PADs [12]. To decrease the number of PADs and in line with our initial studies, we decided to summarize the initial PADs set using the MMRFS technic. The method is described in a previous publication [3]. MMRFS aims to generate a subset of pharmacophores characterized by discriminating, distinct, and representative elements of the active molecules. 135 PADs (see Table 1) out of the 377 initial PADs were selected in this case with a coverage, for the data set, of 77% (instead of 81% without MMRFS selection). Most of the PADs have order 3, corresponding to three pharmacophoric functions (see Table 1). As described in our previous publication, we focused, for the network, on the nearest neighbors of each PAD. The neighbors of each PAD were ranked in descending order based on similarity coefficient values. Using this method, the nearest two neighbors of each pharmacophore were retained (we also analyzed the nearest five and ten neighbors, but the nearest two neighbors were best for the analysis of the network). The two neighbors corresponded to the minimum number of neighbors because several of the edges within a given network can exhibit identical values for the similarity coefficient. We have chosen the Compound Spring Embedder [23] for the layout (PAD network) in Cytoscape [24]. The final PAD network allows us to get a view of our data set (see Fig. 9) with active PADs in solid red and inactive PADs in solid cyan.

**Fig. 10:**
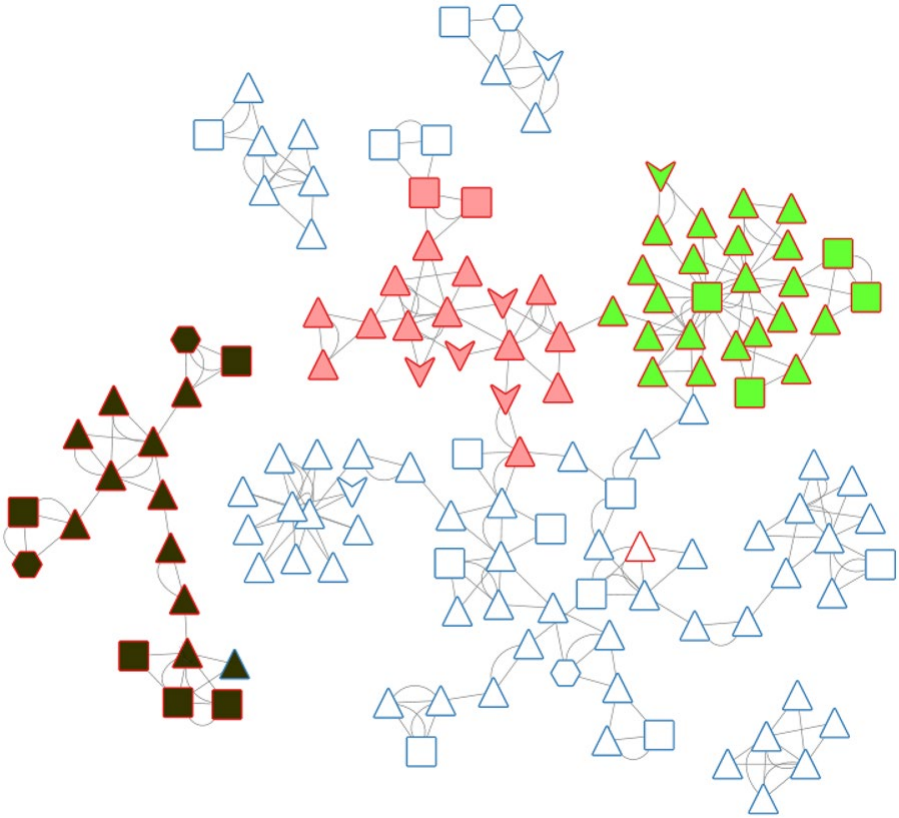
PAD network with the three areas (green, pink and black) for active pharmacophores

From this PAD network, we can distinguish, at first glance, three areas for active PADs (see Fig. 10). A description of the PADs (in brackets, the number of associated chemicals) for these three areas and their representative compounds are provided in Tables 2, 3 and 4. The first one, in solid green, groups 26 PADs and covers 467 molecules (442 actives) with a recall value of 0.57 (57% of all actives). The second one, in solid pink, groups 19 PADs and covers 179 molecules (170 actives). The last area, in solid black, is more isolated. It groups 18 PADs, 17 actives and one inactive. The 17 active PADs cover 175 molecules (160 actives).

**Table 2:**
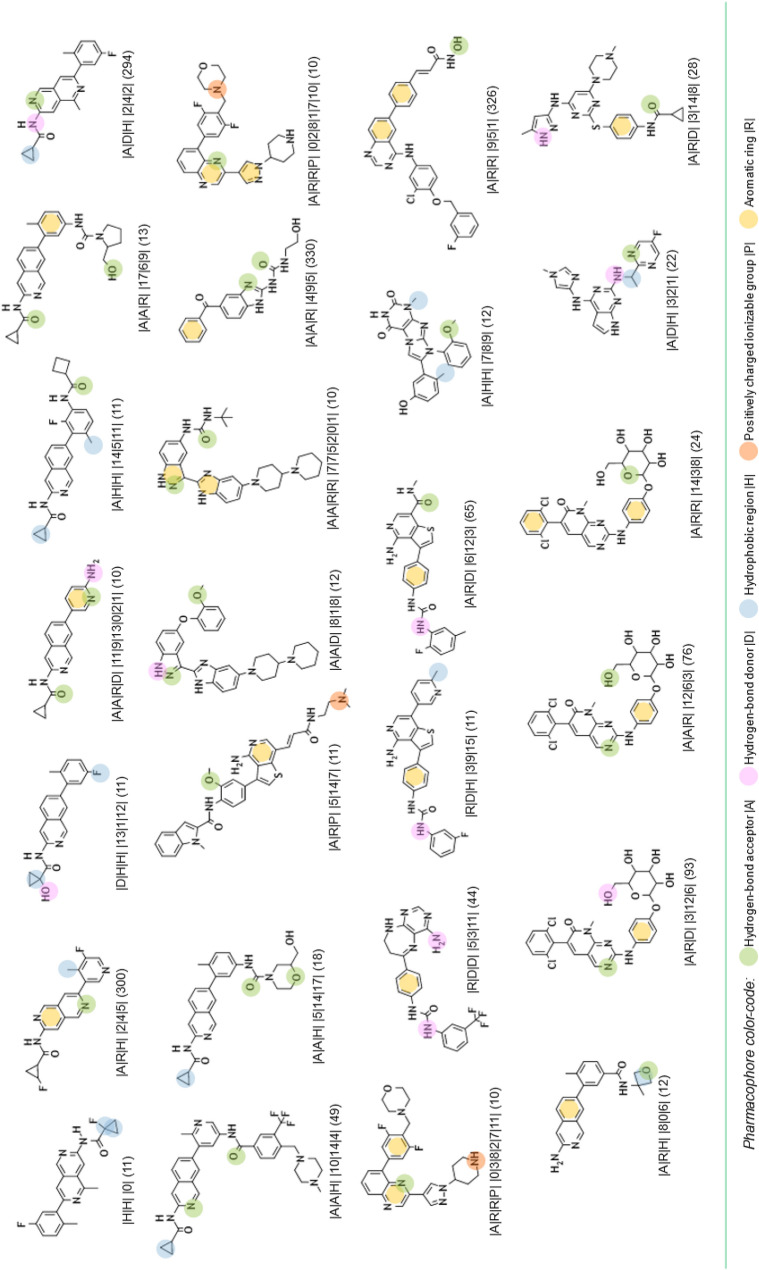
Description of the representative compounds (centroid, ECFP4/Tanimoto) associated to each pharmacophore (with the alignment between molecules and pharmacophores) in the green area and in brackets, the number of associated chemicals

**Table 3:**
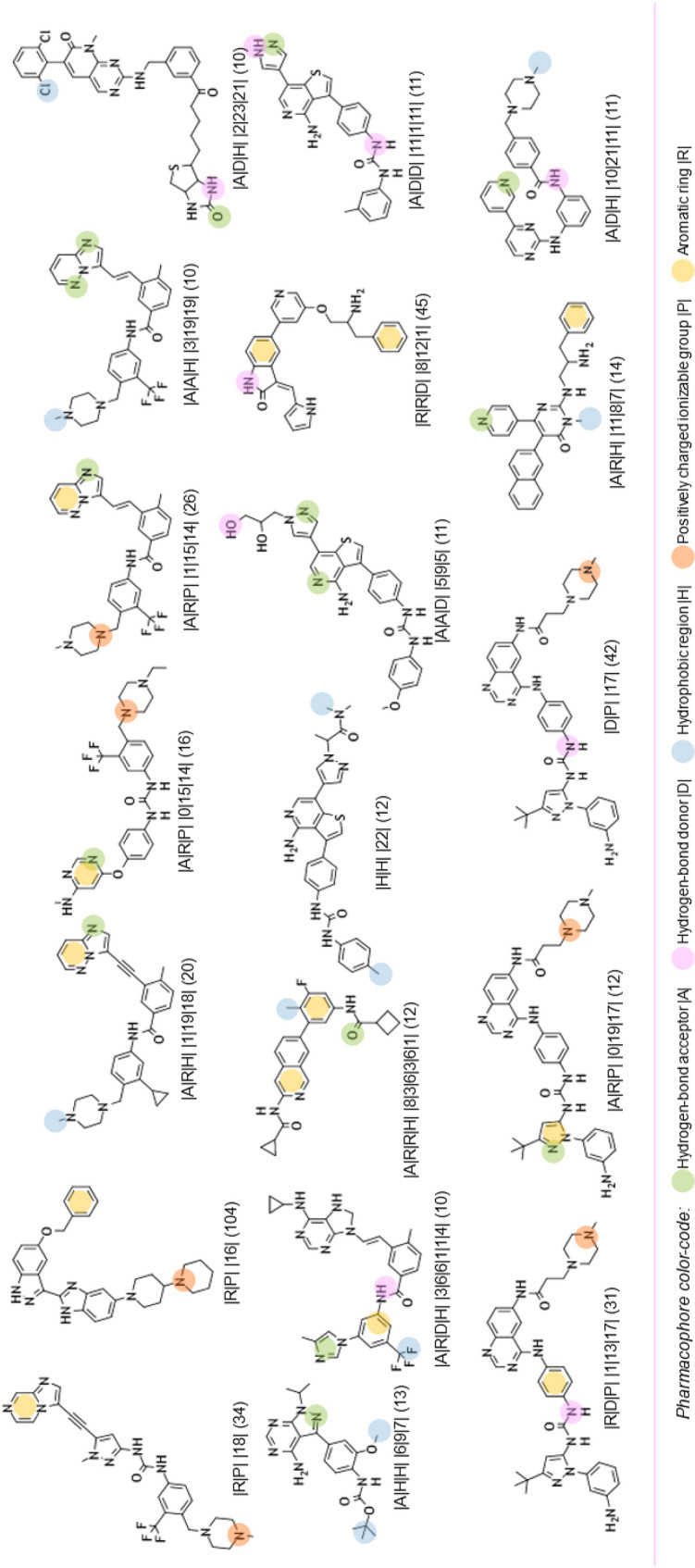
Description of the representative compounds (centroid, ECFP4/Tanimoto) associated to each pharmacophore (with the alignment between molecules and pharmacophores) in the pink area and in brackets, the number of associated chemicals

**Table 4:**
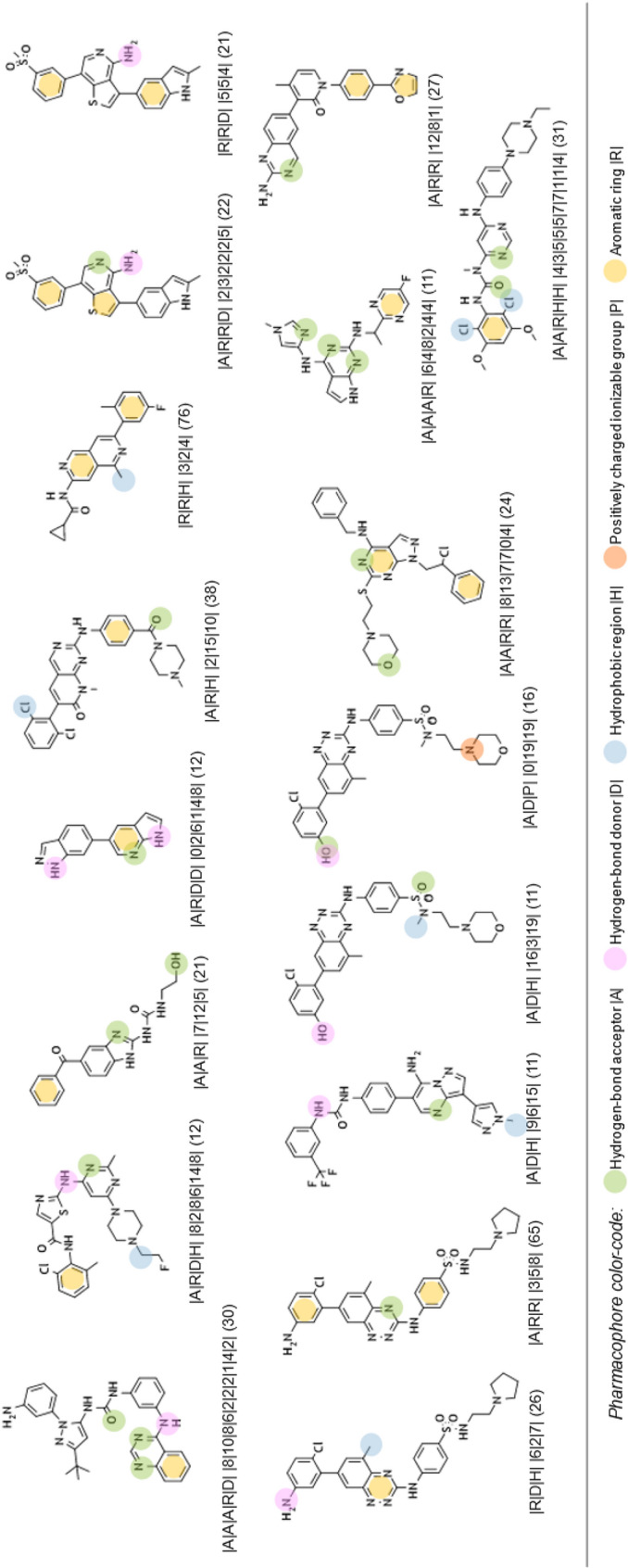
Description of the representative compounds (centroid, ECFP4/Tanimoto) associated to each pharmacophore (with the alignment between molecules and pharmacophores) in the black area and in brackets, the number of associated chemicals

**Fig. 11:**
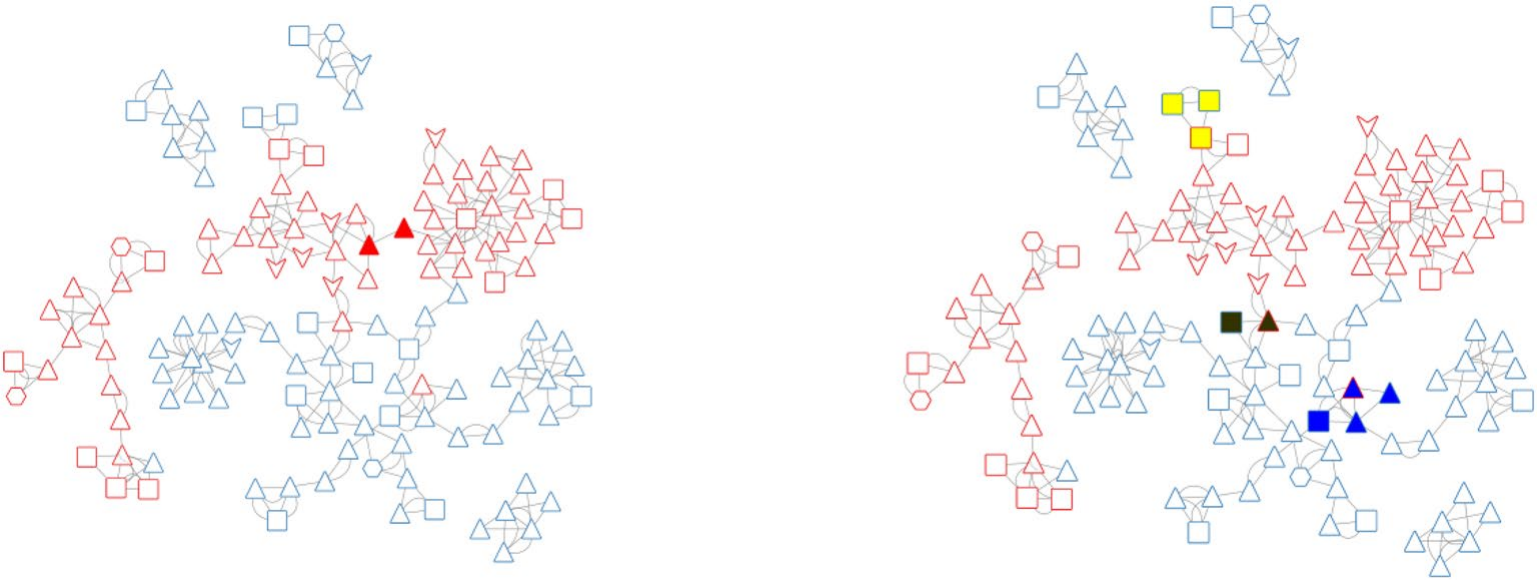
PADs between two active groups (left, solid red) and proximities between active and inactive pharmacophores (right) corresponding to three areas (solid yellow, green, blue)

It is impossible in this publication to describe all the PADs. We have therefore chosen to focus on some specific areas of this PAD network. The first one concerns a connection between two active areas. In fact, the green area is connected to the pink area by two PADs (similarity of 0.39 between the two PADs; see Figs. 10 and 11 (left), solid red).

**Fig. 12:**
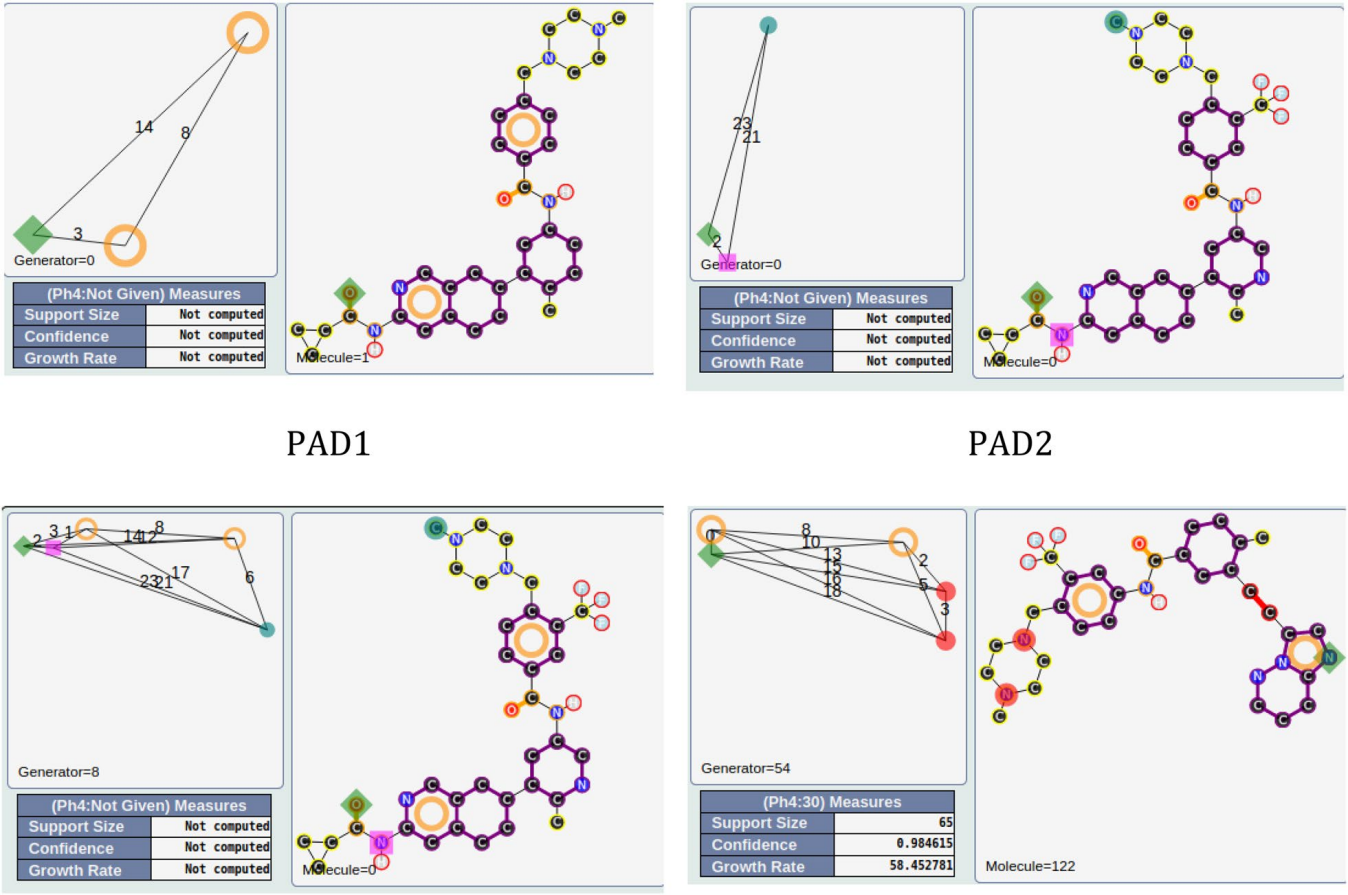
PAD1 and PAD2 with two representative compounds (top). Below, pharmacophore **1** corresponding to a combination of PAD1 and PAD2 (left) and pharmacophore **2** (with ponatinib) derived from the nine compounds fitting pharmacophore **1**

One of these two PADs has 24 compounds (PAD1, |A|R|R| |14|3|8|; see Fig. 12), and the other has 10 compounds (PAD2 |A|D|H| |2|23|21|; see Fig. 12). They share 9 compounds (90% of the compounds associated with PAD2 are in PAD1). By combining PAD1 and PAD2, pharmacophore **1**, with 5 pharmacophoric features, can be derived (see Fig. 12). To analyze the possible proximity of other scaffolds to these nine compounds, we derived all the pharmacophores with 5 features from these nine compounds and extracted those associated with the maximum number of derivatives. Among the 56 pharmacophores generated, the best one (in terms of the number of compounds) is associated with 65 chemicals (pharmacophore 2, GR = 58) and is related to the ponatinib-like family [25].

**Fig. 13:**
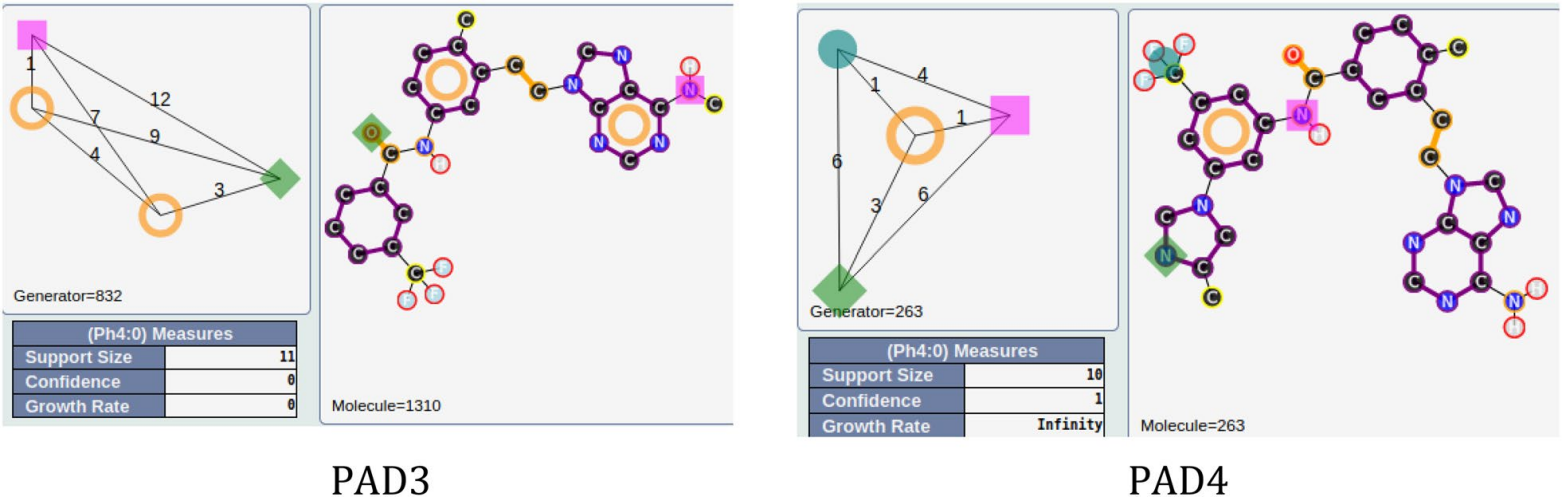
PAD3 (only inactive compounds) and PAD4 (only active compounds) showing the inversion of the amide function (between two aromatic rings) for the two representative compounds of each PAD

For the other areas, we analyzed the situation where active PADs are close to inactive ones (similarity ≥ 0.3 for the PADs). This is the case for three areas (solid yellow, green, blue; see Fig. 11). The first one, in solid yellow, allows us to understand the importance of one |A| function included in an aromatic group and a specific position of the |D| function for similar compounds. In fact, for PAD3 with only inactive compounds, we observed (see Fig. 13) for the representative compound the inversion of the amide function compared to the representative compounds of PAD4 (with only active compounds), and moreover, one |A| function is missing compared to the representative compound of PAD4. PAD4 is the pharmacophore clearly associated with the nilotinib-like family [26].

**Fig. 14:**
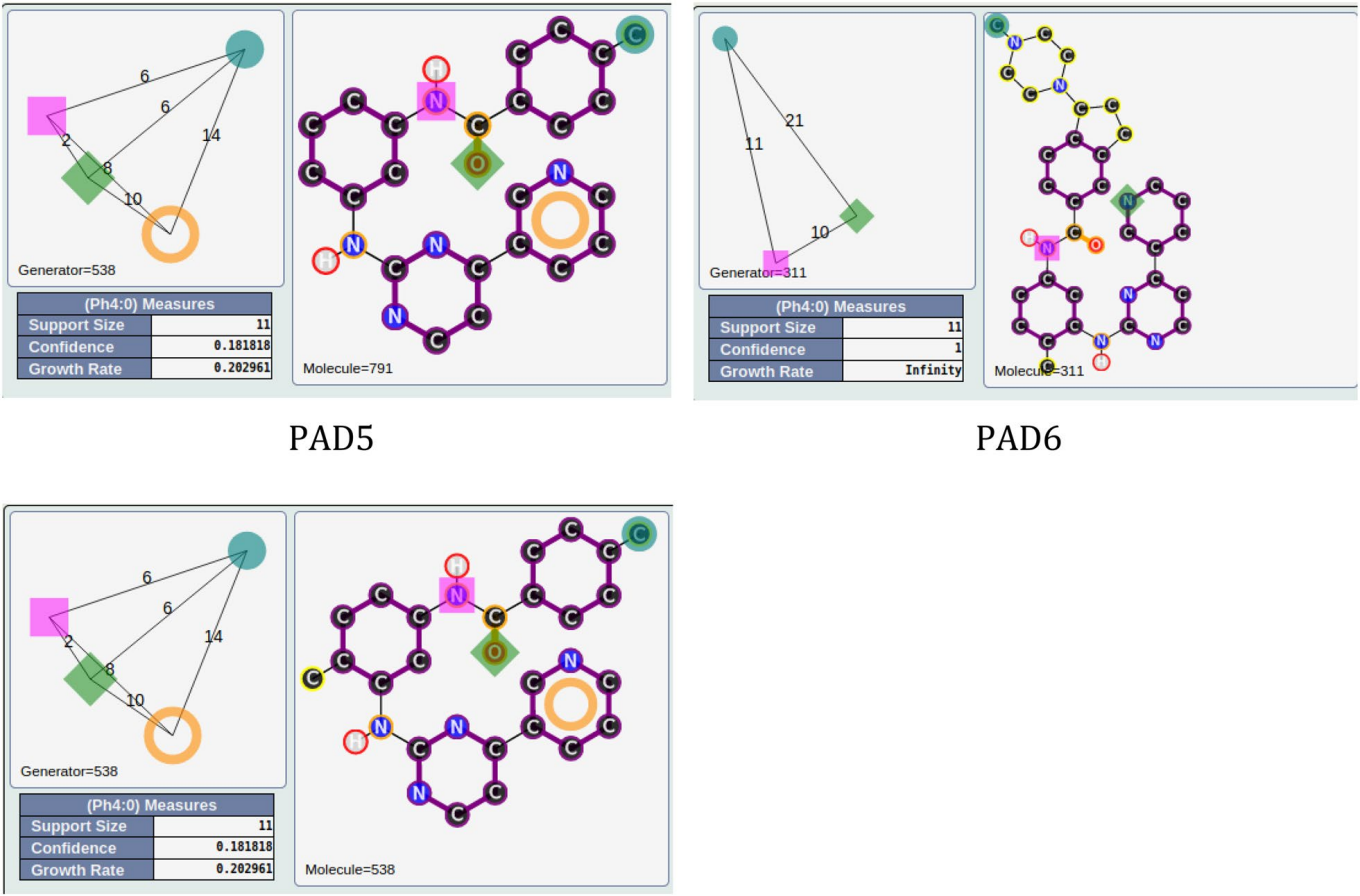
PAD5 and PAD6 with two representative compounds (top). PAD5 with the only active compound (bottom) associated to this PAD and for which the key methyl group is present

The solid green area is associated with pharmacophoric variations around the scaffold associated with imatinib [27]. PAD6 (see Fig. 14) has three pharmacophoric functions translating the position of two polar functions (|A| and |D|) and, above all, the size of the compound with a hydrophobic group being at a distance of 19 (19 edges) from the aromatic ring bearing the |A| function. PAD5 is, on the contrary, associated with inactive derivatives. We observed with PAD5 the typical scaffold associated with imatinib without the terminal amine functions. We can notice that one compound fitting PAD5 is active with the typical methyl group for some kinase inhibitors of ABL1 related to the back pocket binding site [12], also described previously with the best parent (see Fig. 8).

**Fig. 15:**
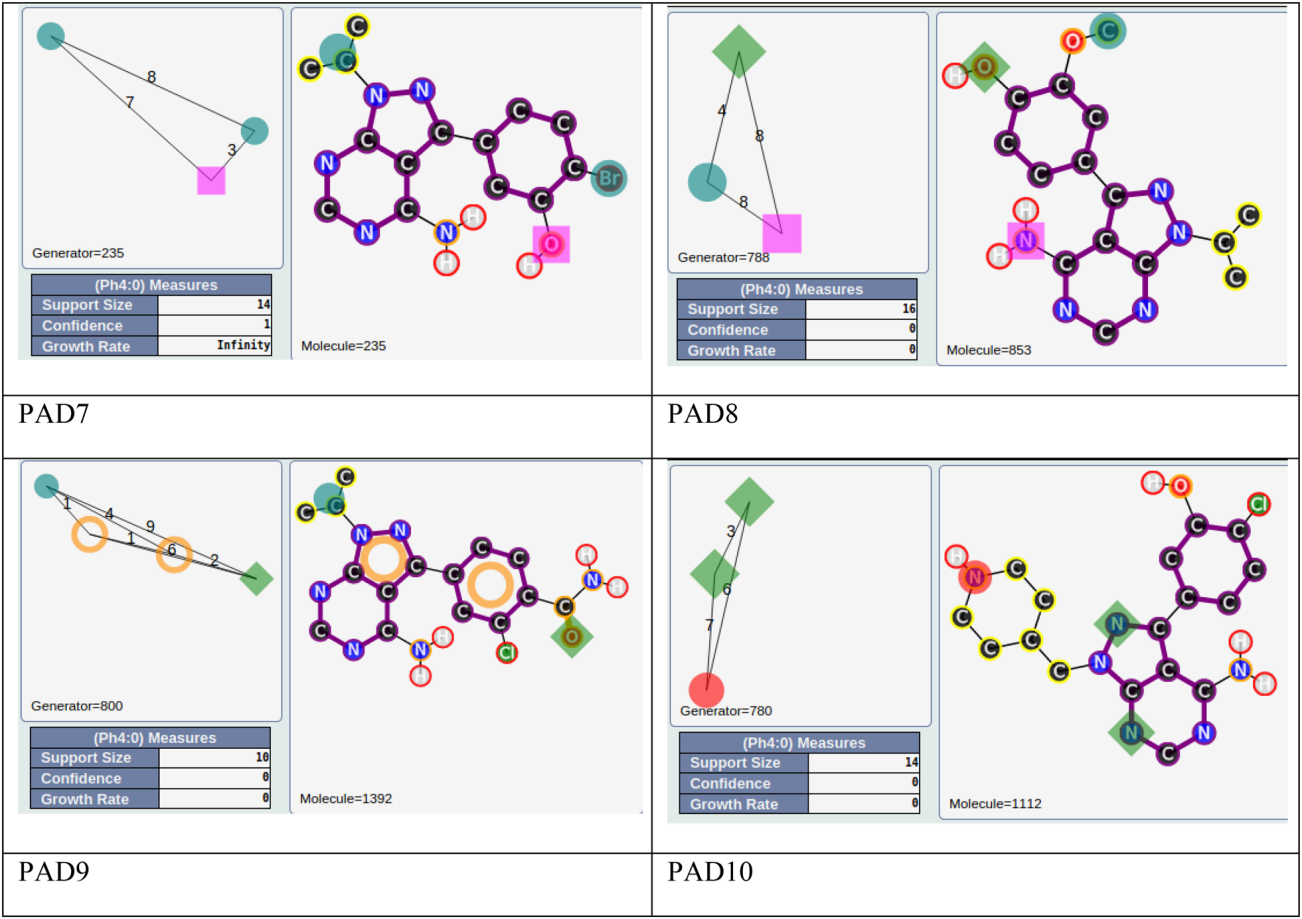
PAD7, PAD8, PAD9, PAD10 with representative compounds. PAD7 is active and the other ones are inactives. PAD7 and PAD8 have different positions of the hydroxy group for phenol functions. No phenol group in PAD9 compared to PAD7. PAD10 with a polar function (amine) instead of a hydrophobic function for PAD7

The last solid blue area is associated with the only active PAD surrounded by inactive PADs (see Fig. 9). PAD7 is active, and the other ones are inactive (see Fig. 15). For this chemical series, we can clearly see the importance of the |H| function in alpha position to the |A| function of the phenol group (PAD7). For PAD8, always on the phenol group, the |H| function is not in the same position (methoxy group in this case). For PAD 9, we do not have a phenol group and the |A| function is in a different position. For PAD10, a |P| function is present. So, some clear structure–activity relationships could be identified from the analysis of these PADs.

### Cross-validation studies and stability of PADs

We performed a stratified tenfold cross-validation study (i.e. each fold contained the same proportions of active and inactive compounds) on the initial dataset (using scikit-learn/Kfold [28]). The method (extraction of pharmacophores and definitions of pharmacophore network/SECs/PADs) was applied independently to each subset.

As implied by, and supporting, our earlier explanation, we extract on average 15.6% fewer pharmacophores (5.7%-31.1%). The number of SECs varies less, between 7.75 and 13.5% fewer, for an average of 10.9% fewer SECs. A total of 364 active PADs were obtained by combining the result derived from the 10 subsets, fewer than the 377 PADs derived from the full data. The method was found to be more stable than we expected. Indeed, of these active PADs, 61% are present in at least 5 subsets (as compared to the 55.49% of *pharmacophores* one would expect to reoccur 5 times) and 26 PADs are present in the results of *all* the subsets. Of these 26 active PADs, |D|A|R| |2|3|2|, with 470 compounds, is associated with the highest number of compounds. Inactive PADs are less stable, with 40% present in at least 5 folds, and 14 PADs are present in all the folds (Table 5).

**Table 5:** Number of pharmacophores (Phar.), SEC and PADs for each fold. Cumulative presence of the PADs in the folds

	F1	F2	F3	F4	F5	F6	F7	F8	F9	F10
Phar	98,053	92,727	77,346	92,742	105,875	95,596	108,556	90,708	91,588	93,638
SEC	14,257	14,278	13,836	13,717	14,058	13,529	13,378	13,415	13,547	13,791
PADs	153/199	168/173	174/144	152/122	130/126	117/149	126/148	130/208	127/180	115/169
*Presence of the PADs in the folds (cumulative: at least x folds)*										
x fold	10	9	8	7	6	5	4	3	2	1
Pertinence ≥ 1.64	26	70	111	146	192	225	261	304	349	364
Pertinence ≤ -1.64	14	33	60	101	139	194	221	268	355	481

## Conclusions

**Fig. 16:**
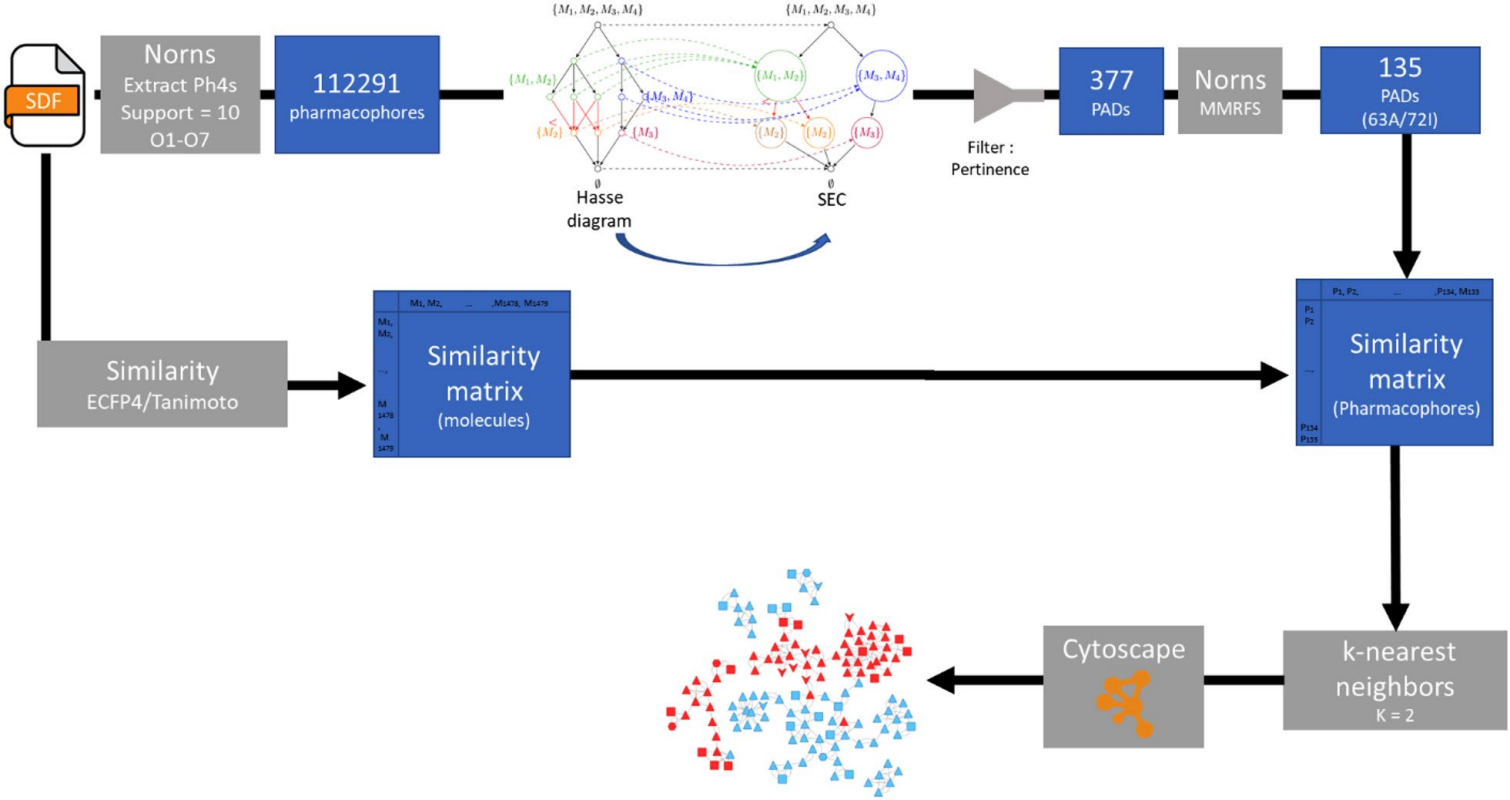
Workflow associated to the different steps of our process from the extraction of pharmacophores to the representation of a network associated to the PADs

In an effort to develop a tool that can rapidly provide information from a dataset of molecules regarding active or inactive compound characteristics, we conducted structural elucidation using a fully annotated dataset of molecules extracted from the ChEMBL database. The various steps involved in this workflow are summarized in Fig. 16. The extraction of pharmacophores with Norns is the most time-consuming process, taking several minutes with our configuration. We processed 1479 molecules to generate topological pharmacophores containing 1 to 7 motifs, with the support of at least 10 molecules. As part of our objective to involve a human expert in pharmacophore elucidation, we established a specific method to identify outstanding pharmacophores known as PADs.

The extraction of PADs is initially linked to defining the 15,477 SECs from the initial 112,291 pharmacophores. Subsequently, calculations of GR_N_ were performed for each SEC. A threshold for the pertinence values associated with each SEC led to the extraction of 377 PADs. In the end, a PAD network was constructed using Cytoscape starting from a representative set of 135 PADs (MMRFS). This network incorporates the similarity between the PADs for link definition (the 2NNs of each PAD).

The interestingness of this reduced set of 135 PADs is based on the diversity of information it provided, equally shared between active and inactive compounds. Cross-validation studies can be also a basis for the selection of interesting PADs. The proximity between these PADs allows us to explain some key SARs with the four illustrations in this publication.

## Data Availability

The datasets supporting the conclusions of this article and the main programs related to this work are available at https://osf.io/pj8n3/?view_only=f49d5a0af5114b568f327c11d46bdfd3. The main program and the sources (https://hal.science/hal-04057516) are available on GitHub: https://github.com/Etienne-Lehembre/Pharmacophores-Activity-Delta. Image_Dockers for Norns are available on docker hub (greyc/norns, https://hub.docker.com/r/greyc/norns). Pipeline Pilot (BIOVIA Pipeline Pilot, Release 7.5, San Diego: Dassault Systems) is commercial software.

## Abbreviations

EP: Emerging Pattern
GR: Growth Rate
MMRFS: Maximal Marginal Relevance Feature Selection
EC: Equivalent Class
GEC: General Equivalent Class
DEC: Divided Equivalent Class
SEC: Structured Equivalent Class
PAD: Pharmacophore Activity Delta
SAR: Structure–Activity Relationships
